# Extracellular Vesicles Mediated Early Embryo–Maternal Interactions

**DOI:** 10.3390/ijms21031163

**Published:** 2020-02-10

**Authors:** Alessandra Bridi, Felipe Perecin, Juliano Coelho da Silveira

**Affiliations:** Department of Veterinary Medicine, Faculty of Animal Sciences and Food Engineering, University of São Paulo, 13635-900 Pirassununga, SP, Brazil; alessandra.brid@usp.br (A.B.); fperecin@usp.br (F.P.)

**Keywords:** small extracellular vesicles, embryo, crosstalk, oviduct, endometrium, blood circulating exosomes

## Abstract

Embryo–maternal crosstalk is an important event that involves many biological processes, which must occur perfectly for pregnancy success. This complex communication starts from the zygote stage within the oviduct and continues in the uterus up to the end of pregnancy. Small extracellular vesicles (EVs) are part of this communication and carry bioactive molecules such as proteins, lipids, mRNA, and miRNA. Small EVs are present in the oviductal and uterine fluid and have important functions during fertilization and early embryonic development. Embryonic cells are able to uptake oviductal and endometrium-derived small EVs. Conversely, embryo-derived EVs might modulate oviductal and uterine function. In this review, our aim is to demonstrate the role of extracellular vesicles modulating embryo–maternal interactions during early pregnancy.

## 1. Introduction

In mammals, the perfect embryo–maternal communication is necessary to allow the establishment and maintenance of pregnancy. For this, oocyte maturation occurs in the preovulatory follicle, followed by the fertilization in the oviduct, and early development in the oviduct and uterus during the luteal phase [1]. Upon ovulation, the oocyte starts its journey through the oviduct, is fertilized, and embryo development begins, followed by its first cleavages [2]. Importantly, the major embryonic gene activation (EGA) occurs during the embryo passage through the oviduct [3]. During this phase, the embryo starts to transcribe more actively, decreasing its dependency on the maternal mRNA stock. The epithelial line of the oviduct is formed by ciliated and secretory cells. These cells play roles in gamete transport, capacitation and fertilization [4,5], and on early embryonic development [6,7], mostly by oviductal secretions [8]. Small extracellular vesicles (EVs) are present in oviductal fluid content and participate in these important reproductive events [5,7].

The embryo enters the uterus 4 to 5 days after oocyte fertilization, in bovine [2,9]. During embryonic development, the embryo reaches the blastocyst stage composed of two cell lines: the inner cell mass that will originate the embryo proper and the trophectoderm cell monolayer, which ultimately will form the fetal adnexa/placenta [2,10]. Fetal appendages will establish contact with the endometrium, and thus establish the interchange interface between embryo/fetus and maternal tissues. However, in order to allow embryo development and placentation, uterine epithelial and glandular cells secrete the uterine fluid that is very important for embryo nutrition because it contains proteins, lipids, amino acids, growth factors [11], and small EVs [12,13,14].

In most domestic mammalian species, the embryo—when in the uterus—increases the secretion of biological molecules related to the maternal recognition of pregnancy (MRP). These molecules have luteotrophic and anti-luteolytic actions, guaranteeing the corpus luteum (CL)’s capability to secrete the progesterone necessary to establish and sustain pregnancy [15,16]. Additionally, different biological molecules are secreted by the embryos of several mammalian species during MRP as interferon-tau (IFNT) in ruminant [17], estradiol in pigs [18], and chorionic gonadotropin in humans [19]. However, recent reports indicate that the embryo is also able to secrete EVs [20,21,22], but their role in MRP is still elusive. 

Therefore, in mammals, the embryos and the female tract (oviduct and endometrium) are able to secrete extracellular vesicles during the pre-implantation period. Extracellular vesicles are cell-secreted vesicles that are classified according the their size, biogenesis, and secretion, as exosomes, microvesicles, and apoptotic bodies [23]. Several cellular types can secrete EVs including follicular [24,25], oviductal, and endometrial cells [13,21,26], as well as in vitro and in vivo produced embryos [20,21,22,27]. Extracellular vesicles carry bioactive molecule as miRNAs, mRNAs [28], proteins [29], and lipids [30], which modulate various reproductive processes such as ovarian follicular development, oocyte maturation, embryonic development, maternal–embryonic communication, and the establishment of mammalian gestation. There is experimental evidence that EVs participate in intercellular communication in essential reproductive events related to the establishment of pregnancy, such as cell proliferation, crosstalk between the maternal organism and conceptus, as well as during embryonic implantation [31]. In early stages of pregnancy, the communication between the conceptus and maternal organism is necessary and the evidence of the participation of EVs of maternal or embryonic origin is increasing [14]. 

Herein, we will review the current knowledge on embryo–maternal interactions, with special emphasis on the roles of EVs during the early crosstalk between embryonic and maternal tissues. 

## 2. Embryo–Maternal Interactions Mediated by Embryotropins

Embryotropins are bioactive molecules such as proteins, lipids, and miRNAs secreted from both in vivo—or in vitro—produced mammalian embryos [32]. These molecules may act in autocrine and/or paracrine ways, modulating the embryo’s development (in vitro culture) and the maternal endometrial cells, respectively [32]. 

In domestic ruminants, the mostly known and well-characterized embryotropin is the IFNT. IFNT is a cytokine secreted by the trophoblastic cells of the ruminant embryo and conceptus that can act in a paracrine and endocrine way. In the uterus, IFNT acts in a paracrine way, decreasing estrogen and oxytocin receptor expression in the endometrium, which is an essential step to maintaining a viable corpus luteum and producing progesterone [33]. Besides antiluteolytic function, IFNT has endocrine effects, stimulating the expression of IFN-stimulated genes (ISGs) in the endometrium [34], in the corpus luteum [35,36,37], in white blood cells [38], and in the liver [39]. The effects of IFNT secreted by the bovine embryo in the maternal organism can be detected as early as day 7 of development [40,41]. 

The IFNT function on the maternal recognition of pregnancy is well established for ruminants. There are parallels for such a response in other species. One example is the conserved response of interferon-stimulated gene 15 (*ISG15*), which is stimulated by IFNs and other cytokines. *ISG15* is up-regulated in the endometrium of ruminants [34], primates [42], and mice [43] during early pregnancy. However, its function during the maternal recognition of pregnancy in non-ruminant species is not well established. Mouse knockout for *Isg15* results in 50% fetal loss, which can be explained by change decidual gene expression that is functionally related to cell survival and adhesion pathways [44]. 

Besides IFNT, day 13 bovine embryos can also secrete prostaglandins, such as prostaglandin F2 alpha (PGF), prostaglandin E2 (PGE2), and prostaglandin I2 (PGI2) [45]. These prostaglandins act in a paracrine way in the endometrium, increasing *ISGs’* expression and function, which can be important for uterine receptivity as well as conceptus growth and development during early pregnancy [45]. In addition, PGE2 and PGI2 can modulate blastocyst implantation, decidualization, and endometrial vascular permeability during early pregnancy in mice and rats [46]. In large domestic species, PGE2 is secreted by the endometrium and embryo, showing an important role as the local antiluteolytic factor (reviewed by [47]). PGE2 secreted by equine viable embryos during early embryonic development is involved with the initial oviductal transport of the embryo and opening the uterutubal papilla to allow the embryo to enter the uterus. This mechanism may explain why unfertilized oocytes or degenerate embryos are not able to gain the uterine lumen in equine [48,49]. Additionally, the lysophosphatidic acid (LPA) is secreted by the bovine embryo and endometrium and may act as a luteotropic factor by stimulating PGE2 synthesis in stromal cells during early pregnancy [50]. 

Hence, embryotropins are signaling molecules acting in the maternal tissues to improve embryo–maternal recognition in mammals. Several embryotropins are secreted by different species with the same aim, which is to inform embryo presence to the mother and to maintain a functional CL, progesterone synthesis, and the maintenance of pregnancy. However, other mechanisms can be involved in the maternal recognition of pregnancy. Recently, EVs were introduced as new players in the embryo–maternal communication.

## 3. Extracellular Vesicles Derived from Female Reproductive Tract and Embryo

Extracellular vesicles (EVs) are formed by a phospholipid bilayer and secreted by different cell types [51]. EVs were discovered approximately 40 years ago, and they were first characterized as “small trash bags”, due to the possibility of carrying cellular material to the extracellular environment [52]. Currently, these EVs have been found in different body fluids and can be divided into exosomes, microvesicles, and apoptotic bodies [23] according to their size, biosynthesis, and contents.

Exosomes are nanovesicles between 30 and 150 nm in size [53] that carry bioactive material such as lipids [30], proteins [29], RNAs, and miRNAs [28], which are able to control regulatory pathways associated with physiological or pathological functions among neighboring or distant cells through the extracellular environment [13]. The biogenesis of small EVs initiates with plasma membrane endocytosis, which gives rise to multivesicular bodies (MVBs). Inside MVBs intraluminal vesicles are formed, which are released out of the cell when the MVB fuses with the cell plasma membrane [54]. The released vesicles are called exosomes (small extracellular vesicles).

Cellular communication mediated by small EVs can happen by fusion between the vesicle membrane and plasma membrane of the target cell, leading to the release of EVs contents into the target cell cytoplasm [13]. In addition to cell fusion, it is suggested that specific receptors present in the membrane of the target cells are involved in EVs’ endocytosis [55,56]. 

Small EVs have also been identified in many body fluids as follicular fluid [24], blood [57], cerebrospinal fluid [58], urine [59], oviduct [7], and uterine fluids [13,21]. In addition to body fluids, embryos cultured in vitro can secrete small EVs in the culture medium [20]. There are different methods to isolate EVs from body fluids and culture medium such as ultracentrifugation, filtration, size-exclusion chromatography, polymer precipitation, immunoaffinity, and microfluidic techniques [60]. Thus, based on the research and type sample, the best isolation method can be chosen [60]. This decision is normally based on the molecule of interest since each method can yield different concentrations and purity. For example, our laboratory has used filtration and ultracentrifugation to isolate small EVs from different body fluids such as follicular fluid [25,61], polymer precipitation, and ultracentrifugation to separate small EVs from an in vitro embryo-conditioned medium [62]. Similarly, other studies have isolated EVs from embryos or conceptus-conditioned culture media using polymer-based precipitation [21] and ultracentrifugation [20,63]. 

Small EVs are involved in several reproductive events related to oocyte maturation, fertilization, early embryonic development, and crosstalk between embryo and maternal organism (Table 1). One of the first studies that identified the presence of EVs associated with reproductive events used equine ovarian follicular fluid to investigate the role of extracellular vesicles during follicular development. The authors demonstrated that EVs from equine ovarian follicular fluid carried miRNAs and proteins, and were uptaken by granulosa cells in vitro and in vivo, suggesting a new cell-to-cell communication inside the ovarian follicles [24]. Another study demonstrated that oviductal microvesicles were uptaken by oocyte cytoplasm after 72 h of maturation in bitch, demonstrating the role of EVs in improving the oocyte maturation rate [64]. Furthermore, another study using murine oviductal EVs demonstrated that EVs carrying plasma membrane Ca^2+-^ ATPase 4 (PMCA4) were uptaken by sperm, thus inducing sperm capacitation prior to fertilization [5].

## 4. Embryo–Maternal Interactions through Oviductal EVs

Oviduct used to be considered just a tubular connection between the ovary and the uterus where the oocyte and sperm passed through [73]. However, the oviduct is composed by ciliated and secretory cells that secrete oviductal fluid [8]. Several studies demonstrated the important biological role of the oviductal fluid during sperm capacitation [74], fertilization [4], and the outset of embryonic development [6]. Additionally, oviductal fluid contents include extracellular vesicles that have an important role during oocyte fertilization [5] and early embryonic development [7]. 

Recently, the functional effects of the EVs derived from the oviduct on gametes and embryos were summarized by Almiñana and Bauersachs (2019) [75]. In this review, we highlight the key findings related to EVs derived from oviductal cells and their effects in early embryonic development. 

EVs from bovine oviduct epithelial cells (BOEC) were used in in vitro embryo production and demonstrated to improve embryo quality based on the increase in the number in trophectoderm and total cells and survival after vitrification [7]. In addition, space-specific EVs secreted from isthmus oviductal fluid were able to increase the survival rate and improve the development as well as the quality of in vitro produced blastocysts [67]. In vitro embryos were able to uptake EVs derived from in vivo oviduct epithelial cells, and this communication benefits the embryo blastocyst rate, survival, and quality [66]. Moreover, EVs secreted by donor oviductal cells increase birth rates after embryo transfer in mice due to decreased apoptosis and improved cellular differentiation in embryos [68]. Altogether, these data show the importance of embryo–maternal interactions mediated by EVs derived from the oviduct during early embryonic development, leading to improved embryo quality and successful pregnancy.

An important problem that may occur during the passage of the zygote through the oviduct is ectopic pregnancy (EP), which occurs when the embryo after fertilization implants outside of the uterine cavity due to structural abnormalities in the fallopian tubes, for example [76,77]. Approximately 1.5%–2% of all the pregnancies are ectopic [78]; 97% are in the fallopian tube (oviduct) (reviewed by [79]). Currently, two diagnosis methods are used to detect EP: measurements of human chorionic gonadotropin(hCG)and progesterone in the serum [77]. The serial serum hCG measurement with intervals of 48 h is needed for the diagnostic; however, during this timecourse, tubal rupture might occur in patients, leading to possible complications in clinical status [77,80]. Therefore, only these two biomarkers are not precise and efficient to detect EP. Recently, new approaches showed that circulating miRNA miR-323-3p can be associated with serum hCG and progesterone to improve EP diagnostic [81]. Thus, the presence of miR-323-3p in serum could serve as a marker for EP. However, more studies are necessary to demonstrate if this miRNA is carried by small EVs or not, in order to become a reliable diagnostic marker.

## 5. Embryo–Maternal Interactions between Uterus and Embryo Mediated by EVs

The mammalian uterus is designed to allow sperm transport [82] as well as embryonic and fetal development [12]. In bovine, the morula stage embryo will arrive at the uterus at the uterotubal junction portion around Day 5 of embryo development [2]. Uterine fluid, termed histotroph in ruminants, is the result of glandular cells secretion inside of the uterine lumen [11,83]. This fluid is very important for the nutrition of the embryo since it contains proteins, lipids, amino acids, growth factors [11], and recently described extracellular vesicles that carry bioactive substances that are important for the early embryonic development [12,13,14]. During early embryo development, intense crosstalk starts between the embryo and the maternal uterine environment. This communication is necessary to induce the maternal recognition of pregnancy; thus, it is important to understand the role of extracellular vesicles in the embryo–maternal interface. 

During maternal recognition of the pregnancy period, EVs were isolated within the uterine flushing of ewes on day 14 of the estrus cycle were fluorescently labeled with PKH67 and observed inside conceptus trophectoderm cells. This finding demonstrates that EVs are involved in paracrine communication between the endometrium and conceptus during the early pregnancy period [21]. In addition to that, sheep endometrial epithelium can secrete exosomes containing *ovine endogenous jaagsiekte retroviruses* (*enJSRV*) mRNA, which acts on trophectoderm via toll-like receptors (TLR) to induce IFNT production [69]. Trophoblast cells from the conceptus at day 15 and 17 secrete EVs containing IFNT that are able to stimulate ISGs’ expression in endometrial cell culture [22,70]. Furthermore, macrophage-capping protein (CAPG) and aldo-keto reductase family 1, member B1 protein (AKR1B1) proteins are present in EVs isolated from the uterine flushing of pregnant cows on days 15 and 17 of gestation [22]. Besides that, EVs isolated from uterine flushing in the pre-implantation period increase the expression of apoptotic genes (*BAX*, *CASP3*, *TNFA,* and *TP53* transcripts) in endometrial cells [70]. In addition to that, endometrial cells treated with EVs from the post-implantation induced and increase in *vascular cell adhesion molecule 1* (*VCAM*) transcript, indicating the modulation of adhesion molecules [70]. Furthermore, exosomes isolated from uterine flushing obtained from pregnant cows on days 17, 20, and 22 were used to treat trophoblast CT-1 cells and did not induce changes in *IFNT* and *CDX2* mRNA expression, suggesting that the pregnancy period may influence EVs’ contents [70]. Together, this information highlights the EVs biological role during the period of maternal recognition of pregnancy, which may enhance embryo–maternal communication and consequently contribute to the maintenance of pregnancy.

Steroid hormones, such as progesterone and estradiol, can induce changes in the EVs secretion in human endometrial cells [26]. Progesterone, which is secreted by corpus luteum, is necessary to the establishment and maintenance of pregnancy and acts in the elongation and survival of the conceptus [84]. Progesterone induces myometrium relaxation and stimulates the production of mucin 1 (MUC-1), which is a protein that prevents conceptus adhesion to endometrium; thus, it can continue elongating and producing IFNT [85] as well as stimulating histotroph production by endometrial glands [11]. On day 10 to 14 of the estrous cycle in ovine, an increase in EVs secretion by endometrial luminal cells occurs, suggesting that progesterone is responsible for this event [71]. Besides that, EVs from the uterine lumen had miRNAs upregulated by progesterone that were predicted to modulate phosphoinositide 3-kinase/ Serine/threonine kinase 1 (PI3K/AKT), bone morphogenetic protein (BMP), and post-transcriptional silencing by small RNA pathways [71]. These results reinforce that progesterone is very important during the onset of pregnancy because it can modulate the endometrial function and consequently contribute to embryo development. 

Extracellular vesicles are also secreted by endometrium and chorioallantoic membrane cells as well as trophectoderm and maternal endothelial cells from sows on day 20 of pregnancy [72]. MiRNAs and proteins within EVs were able to modulate the angiogenesis pathway within trophectoderm and maternal endothelial cells [72]. Moreover, EVs derived from the porcine trophectoderm are uptaken by maternal endothelial cells and stimulate the cellular proliferation of these cells [72]. Together, these results demonstrate that EVs have an important biological role in conceptus–endometrium crosstalk during the establishment of pregnancy in porcine.

Local vascularization between the uterine horn ipsilateral and the corpus luteum is more prominent during the estrous cycle luteal phase than the contralateral phase, suggesting that the oviduct and uterine could signal to CL and adjacent tissue for future pregnancy and/or luteal vascularization maintenance [86]. This elevated vascularization on the ipsilateral horn could be involved with the early onset of pregnancy recognition. As an example, exosomal miRNAs were identified in serum samples of nonpregnant and pregnant mares on days 9, 11, or 13 postovulation [87]. These miRNAs were increased in nonpregnant mares and predicted to target the pathway of focal adhesion molecules (FAMs) in the endometrium [87], which are involved in the regulation of the extracellular matrix [88]. These data suggest that in pregnant mares, FAMs are normally abundant, which suggests that exosomal miRNAs are less necessary to modulate focal adhesion pathway mRNAs in the endometrium, allowing the embryo to move inside the uterus, which contributes to the maternal recognition of pregnancy [87]. 

In bovine, 27 miRNAs were highly abundant in the serum small EVs of cows with embryonic mortality compared to the pregnant group on day 17 [89]. These miRNAs modulate pathways associated with many important processes such as inflammation, cell proliferation, endometriosis, cell cycle progression, contraction, infection, late-onset preeclampsia, apoptosis, differentiation, uterine leiomyoma, ovarian endometriosis, and cell viability [89]. However, in a retrospective study examining EVs isolated from the blood plasma of pregnant cows on day 21 of gestation, a low abundance of 27 miRNAs was identified in samples from initial somatic cell nuclear transfer (SCNT) embryonic loss compared with full-term SCNT pregnancies and full-term artificial insemination pregnancies. These miRNAs modulate the pathways associated with pregnancy establishment as well as cell proliferation, differentiation, apoptosis, angiogenesis, and uterus embryonic development [90]. In addition, 29 miRNAs from serum small EVs were differently detected in the 30 days of pregnancy group compared to the normal group [91]. Different pathways involved in metabolism are modulated by these 21 up-regulated miRNAs and eight down-regulated miRNAs in pregnant cows [91]. 

In Table 2, we itemized the publications about EVs carrying miRNAs in the pregnant female blood. On day 9, in pregnant mares, miRNA-29c and miRNA-101 are down-regulated in circulation exosomes [87]; however, on day 21, in bovine, these miRNAs are more abundant [90]. On day 17, in bovine, miRNA-15a, miRNA-15b, miRNA-101, miRNA-106b, miRNA-652, miRNA-143, and miRNA-148a presented decreased expression [89]; however, on day 21, miRNA-15a, miRNA-15b, miRNA-101, miRNA-106b, and miRNA-652 [90], and on day 30, miRNA-143 and miRNA-148a [91] expression increased in the serum-derived small EVs of pregnant cows. MiRNA-148b is increased in blood exosomes on days 21 and 30 of bovine pregnancy [90,91]. However, on day 21, miRNA-193b is more abundant, but on day 30, miRNA-193b is down-regulated in pregnant cows [90,91].

These data suggest that there is EV-mediated communication between the uterus and peripheral circulation. This communication can be direct and modulated by biological factors secreted by the embryo, or it can be indirect, where the embryo stimulates an endometrium response. Thus, extracellular vesicles can be part of this intricate mechanism improving embryo–maternal interactions and consequently pregnancy success in mammals. Moreover, these studies demonstrate the potential role of circulating exosomal miRNAs as biomarkers in early embryonic mortality or early pregnancy diagnosis.

**Figure 1:**
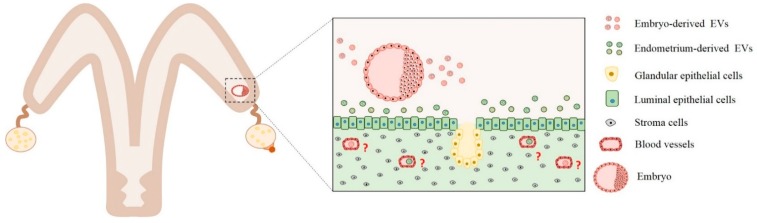
Embryo–maternal interactions mediated by extracellular vesicles secreted from the embryo and endometrium cells. Extracellular vesicles secreted by embryos are uptaken by endometrial cells. Extracellular vesicles (EVs) content include miRNA, mRNAs, and proteins that act by paracrine signaling. Different experiments demonstrated that embryo-derived EVs can modulate interferon-tau, apoptosis, cell proliferation, adhesion, angiogenesis, and cell survival biological pathways. Similarly, endometrium cells can secret EVs containing proteins, miRNAs, and mRNAs. These EVs are uptaken by trophoblast cells, and their contents are predicted to regulate interferon-tau production and angiogenesis pathways. However, questions related to how embryo-derived EVs can exit the uterus and arrive in other maternal tissue cells are yet unclear (red interrogation points). Therefore, further studies are needed to understand how the factors secreted by the embryos exit the uterus and to arrive in other maternal tissue, as blood cells, to modulate biological pathways.

In conclusion, small EVs carry important bioactive molecules that are involved in embryo–maternal crosstalk (Figure 1) through the modulation of important signaling pathways such as angiogenesis, apoptosis, interferon-tau, adhesion, proliferation, and cell survival. The above-mentioned studies demonstrate that EVs participate in embryo–maternal interactions during early embryonic development and the maternal recognition of pregnancy in mammals.

## 6. Conclusions

In this review, we demonstrate the biological roles of extracellular vesicles in events occurring during the onset of pregnancy and involved in the communication between the embryo and the maternal organism in different mammalian species. EVs carry important bioactive molecules that are capable of modulating key reproductive events during the early pregnancy period. Further investigations are necessary to elucidate if EVs secreted by the oviduct and endometrium as well as embryos can arrive in peripheral circulation and modulate different pathways in maternal organisms. Thus, the progression in our understanding related to this type of communication can advance the tests to detect pregnancies, abnormal pregnancies (EP), and predict pregnancy loss, as well as push the development of new technologies to modulate early embryo–maternal interactions.

## Figures and Tables

**Table 1:** Major findings about extracellular vesicles (EVs) derived from the reproductive female tract and embryos in mammals. IFNT: interferon-tau.

Topic	Findings	Reference
**EVs derived from ovarian follicles**	√ Characterization of protein and miRNA content	Da Silveira et al., 2012 [24]
	√ EVs from follicular fluid are uptaken by granulosa cells in vivo and in vitro	
**EVs derived from in vitro produced embryos**	√ In vitro fertilization (IVF) and parthenogenetic embryos secrete EVs	Mellisho et al., 2017 [20]
	√ Small EVs from parthenogenetic porcine embryos improves cloned embryos’ development	Saadeldin et al., 2014 [65]
	√ Exposure of somatic cell nuclear transfer (SCNT )embryos to EVs from other SCNT embryos increases the blastocyst rate	Qu et al., 2017 [63]
**Oviductal EVs**	√ Endocytosis by oocyte cytoplasm improves maturation rate	Lange-Consiglio et al., 2017 [64]
	√ Uptake of EVs by sperm cells modulate sperm capacitation	Al-Dossary et al., 2013 [5]
	√ Improve in vitro embryo quality	Lopera-Vásquez et al., 2016; Almiñana et al., 2017; Lopera-Vasquez et al., 2017 [7,66,67]
	√ Increase birth rates after embryo transfer	Qu et al., 2019 [68]
**Embryonic and uterine EVs**	√ EVs isolated from uterine flushing are uptaken by conceptuses trophectoderm cells	Burns et al., 2016 [21]
	√ Endometrial EVs containing *enJSRV* mRNA act on trophectoderm to induce IFNT production	Ruiz-González et al., 2015 [69]
	√ EVs derived from trophoblast cells contain IFNT that stimulates *ISGs* expression in endometrial cells	Nakamura et al., 2016; Kusama et al., 2018 [22,70]
	√ CAPG and AKR1B1 proteins are present in EVs isolated from uterine flushing	Nakamura et al., 2016 [22]
	√ Progesterone increases the release of EVs from endometrial cells	Burns et al., 2018 [71]
	√ Endometrial-derived exosomes containing miRNAs upregulated by progesterone can modulate phosphoinositide 3-kinase/ Serine/threonine kinase 1 (PI3K/AKT), bone morphogenetic protein (BMP), and post-transcriptional silencing by small RNAs pathways	
	√ Exosomal miRNAs and proteins modulate the angiogenesis pathway in trophectoderm and maternal endothelial cells	Bidarimath et al., 2017 [72]

**Table 2:** The main published papers about extracellular vesicle-associated miRNAs in pregnant female blood.

Species	Period of Pregnancy	Circulating Exosomal miRNAs in Pregnant Female	Pathways Modulated by miRNAs	Reference
Equine	Day 9	Down-regulated: eca-miR-27a, eca-miR-29c, eca-miR-101, and eca-miR-486-5p	Extracellular matrix (ECM)–receptor interaction Proteoglycans in cancer TGF-beta Focal adhesion pathway	Klohonatz et al., 2016 [87]
	Day 11	Down-regulated: eca-miR-195 Up-regulated: eca-miR-767-5p	Regulating pluripotency of stem cells Fatty acid biosynthesis FoxO Focal adhesion pathway	
	Day 13	Down-regulated: eca-miR-188-5p, eca-miR-653, eca-miR-874, and eca-miR-140-3p Up-regulated: eca-miR-30c and eca-miR-323-5p	Glutamatergic synapse Regulation of actin cytoskeleton Long-term potentiation Focal adhesion pathway	
Bovine	Day 17	Down-regulated: bta-let-7c, bta-mir-100, bta-mir-101-1, bta-mir-101-2, bta-mir-106b, bta-mir-125b-2, bta-mir-127, bta-mir-141, bta-mir-143, bta-mir-148a, bta-mir-15a, bta-mir-15b, bta-mir-16a, bta-mir-16b, bta-mir-1839, bta-mir-199a-1, bta-mir-199b, bta-mir-2415, bta-mir-25, bta-mir-331, bta-mir-339b, bta-mir-3596, bta-mir-3604-1, bta-mir-409b, bta-mir-451, bta-mir-652, bta-mir-99a	Cancer Connective tissue disorders Organismal injury and abnormalities Reproductive system disease Endocrine disorders	Pohler et al., 2017 [89]
Bovine	Day 21	Highly abundant: bta-mir-15a, bta-mir-221, bta-mir-425-5p, bta-mir-101, bta-mir-93, bta-mir-106a, bta-mir-106b, bta-mir-22-5p, bta-mir-652, bta-mir-103, bta-mir-138, bta-mir-193b, bta-let-7f, bta-let-7g, bta-mir-15b, bta-let-7e, bta-let-7a-5p, bta-let-7d, bta-mir-660, bta-mir-29a, bta-mir-30d, bta-mir-497, bta-mir-148b, bta-mir-361, bta-mir-30a-5p, bta-mir-29c, bta-mir-29d-3p	Wnt TGF-beta Renal cell carcinoma Melanoma Colorectal cancer Glioma p53 Pancreatic cancer; Bladder cancer Dorsoventral axis formation	De Bem et al., 2017 [90]
Bovine	Day 30	Down-regulated: bta-miR-193b, bta-miR-197, bta-miR-339a, bta-miR-326, bta-miR-484, bta-miR-486, bta-miR-423-3p, bta-miR-92a Up-regulated: bta-miR-146b, bta-miR-27b, bta-miR-26b, bta-miR-200a, bta-miR-450b, bta-miR-199c, bta-miR-194, bta-miR-6119-3p, bta-miR-7, bta-miR-199a-3p, bta-miR-574, bta-miR-215, bta-miR-148a, bta-let-7a-3p, bta-miR-21-5p, bta-miR-126-5p, bta-miR-148b, bta-miR-143, bta-miR-1246, bta-miR-192, bta-miR-98	Membrane trafficking Chromosome and associated proteins Exosome G protein-coupled receptors Transcription factors Ubiquitin system Olfactory transduction Transporters Protein kinases Cytoskeleton proteins Cell adhesion molecules and their ligands Protein phosphatase and associated proteins Peptidases Pathway in cancer Messenger RNA Biogenesis CD molecules PI3K–Akt signaling pathway Human papillomavirus infection Mitochondrial biogenesis Spliceosome	Markkandan et al., 2018 [91]
